# A Qualitative Analysis of the Mental Health Training and Educational Needs of Firefighters, Paramedics, and Public Safety Communicators in Canada

**DOI:** 10.3390/ijerph19126972

**Published:** 2022-06-07

**Authors:** Liana Lentz, Lorraine Smith-MacDonald, David C. Malloy, Gregory S. Anderson, Shadi Beshai, Rosemary Ricciardelli, Suzette Brémault-Phillips, R. Nicholas Carleton

**Affiliations:** 1 Department of Philosophy, King’s University College at Western, London, ON N6A 2M3, Canada; smithmac@ualberta.ca (L.S.-M.); david.malloy@kings.uwo.ca (D.C.M.); 2 Faculty of Science, Thompsons River University, Kamloops, BC V2C 0C8, Canada; ganderson@tru.ca; 3 Heroes in Mind, Advocacy and Research Consortium, Faculty of Rehabilitation Medicine, University of Alberta, Edmonton, AB T6G 2R3, Canada; suzette2@ualberta.ca; 4 Canadian Institute of Public Safety Research and Treatment, University of Regina, Regina, SK S4S 0A2, Canada; shadi.beshai@uregina.ca (S.B.); rricciardell@mun.ca (R.R.); nick.carleton@uregina.ca (R.N.C.); 5 Department of Psychology, University of Regina, Regina, SK S4S 0A2, Canada; 6 Department of Sociology, Memorial University of Newfoundland and Labrador, St. John’s, NL A1C 5S7, Canada

**Keywords:** moral injury, public safety personnel, paramedics, firefighters, public safety communicators, mental health, trauma, education, training

## Abstract

Background—Public safety personnel (PSP) are at heightened risk of developing mental health challenges due to exposures to diverse stressors including potentially psychologically traumatic experiences. An increased focus on protecting PSP mental health has prompted demand for interventions designed to enhance resilience. While hundreds of available interventions are aimed to improve resilience and protect PSPs’ mental health, research evidence regarding intervention effectiveness remains sparse. Methods—Focus groups with PSP elicited a discussion of psychoeducational program content, preferred modes of program delivery, when such training should occur, and to whom it ought to be targeted. Results—The results of thematic analyses suggest that PSP participants feel that contemporary approaches to improving mental health and resilience are lacking. While welcomed, the provision of sporadic one-off mental health and resilience programs by organizations was seen as insufficient, and the available organizational mental health supports were perceived as being questionable. The available programs also left participants feeling insufficiently prepared to deal with personal mental health problems and in discussing mental health concerns with co-workers. Conclusions—Participants reported needing more engaging methods for delivering information, career-long mental health knowledge acquisition, and a systems approach to improve the workplace culture, particularly regarding mental health.

## 1. Introduction

The impact of compromised mental health among public safety personnel ((PSP) e.g., border services, correctional workers, firefighters, paramedics, police officers, public safety communicators (e.g., call center operators/dispatchers)) is considerable. When compared to their civilian counterparts, PSP report elevated rates of problematic mental health symptoms, including suicidal behaviors [1,2]. There is also evidence that PSP experience high rates of burnout [3] and compassion fatigue [4,5]. Mental health challenges appear to be associated with extremely frequent exposures to potentially psychologically traumatic events (PPTE) [6], potentially morally injurious events (PMIEs; i.e., transgressions of morals, ethics, and values) [7,8], and several other occupational stressors [9]. PPTE exposures increase the risk for PSP to experience a posttraumatic stress injury [6,10] and PMIE exposures increase the risk for a moral injury (MI) [11,12]. An increased focus on workplace psychological wellness and resilience is underway to protect PSP mental health [13,14]. This focus has also increased the demand for interventions designed to strengthen resilience [15,16,17]. While hundreds of interventions designed to protect PSPs’ mental health are available, research evidence regarding intervention effectiveness remains sparse [18,19]. The aim of this qualitative study was to increase the understanding of what topics PSP would like to learn about in psychoeducational training, what delivery methods of this education are preferred, and who in PSP organizations are in most need of receiving workplace mental health training.

### 1.1. Resilience

Resilience is considered to be instrumental for supporting psychological well-being in occupations where people are frequently exposed to stressors [20,21]; there is no consensus, however, on the definition of resilience [22]. The lack of a clear and accepted definition and a gold standard measure of resilience contribute to the difficulty of comparing intervention studies and identifying what components may most benefit individuals in their pursuit of enhanced resilience. The most common definition of resilience describes an ability to bounce back and recover from adversity [23]. This definition of resilience may imply a quick fix or rapid recovery from one or more PPTE. For PSP, resilience may be conceptualized broadly as the ability of individuals and organizations to adapt and maintain well-being in their complex occupational environments [24]. The broader definition of resilience describes a multifactorial and modifiable process over time. Questions have been raised about the presumed ability of psychoeducation and interventions to modify personality traits, risk factors, or biological predispositions and therein increase resilience [18,19]. Researchers have underscored the impact of organizational or situational stressors on individual resilience, suggesting that current definitions and models are overly simplistic, and inadvertently blame any lack of resilience entirely on individuals [25]. In contrast, there is evidence that diverse occupational stressors can negatively impact mental health [9]. Resilience training should recognize both trait (unmodifiable) and process (modifiable) conceptualizations of resilience [26,27] within a socio-ecological perspective of resilience [28].

### 1.2. Interventions

Various interventions have been utilized to enhance resilience among PSP. Of particular note are the methods of psychoeducation, cognitive behavioral therapy (CBT), stress management, relaxation training, and multi-dimensional training (combining mindfulness, relaxation, and meditation), each of which will be discussed below.

Psychoeducational interventions (1) are often used as part of proactive, ameliorative, or reactive efforts to enhance individual and collective resilience [15,29]; (2) can be delivered to individuals or groups and be focused on individuals or organizations, as well as be delivered in-person or asynchronously in digital formats [30]; (3) have content that generally draws from cognitive behavioral therapy [15,26], stress management programs [16,31], relaxation training [31], or mindfulness-based training [15]. Cognitive behavioral [31] and mindfulness-based psychoeducational interventions [16] are the most commonly studied approaches to resilience training in general and in PSP workplaces [26,32]. Despite their frequent use, there is relatively little evidence regarding the impact of psychoeducational interventions on PSP resilience [33,34]. The Road to Mental Readiness (R2MR) program has had broad uptake from PSP organizations in Canada [35,36]. R2MR is designed to reduce stigma, establish a Mental Health Continuum Model, and introduce the “Big 4” coping and resilience skills. The content is typically delivered in a 4 h session for front line workers and an 8 h session for supervisors and leaders. Aggregated research results indicate that R2MR for PSP produces statistically significant increases in self-reported or perceived resilience (z = 12.95) and reductions in stigma, with modest impacts and small effect sizes [35]; however, multilevel analytic results from a longitudinal assessment of R2MR demonstrated no sustained statistically significant changes in general anxiety disorder d = −0.20 (95% CI (−0.53, 0.13)), major depressive disorder d = 0.038 (95% CI (−0.48, 0.52)), PTSD d = 0.067 (95% CI (−0.35, 0.61)), resilience d = 0.106 (95% CI (−0.23, 0.45)), or work engagement d = −0.134 (95% CI (−0.52, 0.19)) after twelve months [37].

Cognitive behavioral therapy (CBT) is also commonly used to foster resilience. The tenets of cognitive behavioral therapy (CBT) suggest that psychopathology consists of maladaptive associations among thoughts, behaviors, and emotions [38]. CBT-informed interventions encourage learners to identify, dispute, and constructively change any thoughts, beliefs, assumptions, and emotions that seem to cause them distress [38]. Techniques may include empathic listening, validation, self-monitoring, behavioral activation, value clarification, interpersonal effectiveness, coping strategies, cognitive restructuring, and, at times, relaxation techniques [39]. The components of CBT have been identified as being potentially beneficial tools for increasing individual resilience by minimizing negative thoughts, behaviors, and emotions, as well as promoting adaptive coping and potentially resilient thoughts, behaviors, and beliefs [40]. A meta-analytic project focused on resilience interventions identified four studies which exclusively used CBT and evidenced the standardized mean difference between CBT-based resilience interventions and control groups as being 0.27 (95% CI 0.05 to 0.50), indicating a small positive effect [41]. CBT-based resilience interventions have been implemented in diverse populations such as in persons with chronic illnesses [42,43], people displaying symptoms of major depressive disorder [44], new mothers [45], immigrants [46], children [47], youth [48], college students [49], and military spouses [50]. More recently, CBT has been suggested as being a potentially effective way to build resilience during the COVID-19 pandemic by focusing on reality testing, correcting distorted thinking and dysfunctional beliefs, and supporting people to think and act more realistically [51,52].

Stress management interventions aimed at increasing PSP resilience have been frequently employed, with several systematic reviews having summarized research in this area [16,18,26,30,31]. The available evidence suggests participants report small and time-limited improvements in well-being, subjective mental health outcomes [16,18,26,31], and anxiety [26]. There is almost no evidence of physiological changes (i.e., heart rate, blood pressure, hormone levels) from stress and resilience intervention training programs [31,53,54]. Meta-analytic analyses suggest there is insufficient evidence to demonstrate the effectiveness of stress management interventions for reducing negative physiological, psychological, or behavioral outcomes among PSP [55]. The available evidence is limited by the duration of training and a substantial variability in outcome measures [16,26,30,31].

Relaxation training can include an array of techniques designed to improve wellness [56], decrease physical and psychological tension [57], and to precipitate a restful state [58]. Relaxation training techniques include progressive muscle relaxation (PMR), slow breathing and guided imagery [59], yoga, and music [60]. PMR involves sequentially contracting and releasing different muscle groups in an effort to recognize and discriminate between sensations of tension and relaxation. With practice, PMR can improve the perceptions of muscle tension as an indicator of stress and anxiety [56], and help relax the muscles and therein reduce stress. A systematic review of relaxation interventions evidenced positive effects on symptoms of mood- and anxiety-related disorders in older adults, with PMR training, music intervention, and yoga producing the greatest influence [60]. In firefighters, a 4-week training program consisting of 8 h in-person mindfulness sessions appeared to increase psychological resilience more than relaxation training or no training [61].

Multi-dimensional training that includes relaxation may be more effective in decreasing stress and burnout in PSP [62]. Several recent studies have combined mindfulness, relaxation, and meditation in resilience training for PSP [63]. A meta-analysis of six studies indicated that when administered online or through other low-intensity mediums, meditation or mindfulness-based interventions appeared to produce large effects (g = 0.60, 95% CI 0.34 to 0.85, *p* ≤ 0.00, I2 = 0.0%) for increasing psychological resilience among PSP [32]. Mindfulness-based resilience training (MBRT) has increasingly been used to reduce psychological distress and promote well-being [64,65]. Mindfulness skills include attending to, regulating, and enhancing the curiosity and acceptance of the full gamut of present-moment experiences, anchoring one’s awareness of their physical sensations (e.g., breath, body), emotions, and cognitions [66]. Skill development appears to instigate other mechanisms that can reduce distress and promote resilience such as improved emotional control and self-regulation [67], value clarification [68], body and introspective awareness [69], self-compassion [70], and shifts in self-perspective [71,72]. MBRT and mindfulness-based stress reduction (MBSR) appear to mitigate negative health outcomes through enhanced coping [73], augmented wellness [74], and self-efficacy [55], as well as decreased symptoms of burnout [55,74], negative affect [54], and health complaints [54]. MBRT research with PSP has evidenced positive effects for police officers and law enforcement [55,75,76]. Less MBRT research is available when using data from firefighters [41,55,61,77] or paramedics [78].

The available literature suggests that many interventions may support PSP psychological well-being and resilience. There is a paucity of research, however, regarding which aspects of resilience, wellbeing, and mental health that PSP want to be prioritized, and how PSP want interventions delivered. Identifying the specific content and training modalities can help researchers, program developers, and policy makers maximize uptake, engagement, and impact. The current study was designed to identify PSPs’ preferred modes of delivery for psychoeducational material (e.g., resilience, mental health, moral injury), when and how training should occur, and with what foci such training should have.

## 2. Methods

### 2.1. Study Design

The current article presents the results of a qualitative study that employed a thematic analysis that was conducted on a discussion of the preferred modes of delivery of psychoeducational material with firefighters, paramedics, and emergency communication operators. When such training should occur and to whom the information ought to be targeted was also examined.

### 2.2. Sample

Potential participants were recruited from two provinces in Western Canada via snowball and convenience sampling. Recruitment posters were sent to respective PSP organizations and potential participants were instructed to contact the researchers directly to learn more about the study. The inclusion criteria for participation were being English-speaking; being employed as frontline firefighters, paramedics, police officers, or public safety communicators; having at least one year of experience in their profession. The authors deemed the sample size (*n* =19) was sufficient because data saturation (i.e., no new data was being discovered) was reached. This sample size is also in accordance with the general guidelines of qualitative research [79]. The Research Ethics Board (i.e., University of Alberta Pro #00102000, University of Regina REB# 2020-115, Western University Project ID#115902) and PSP organizational approvals were obtained prior to data collection.

### 2.3. Data Collection

Consent forms and demographic questionnaires (e.g., age, sex, gender, profession, organizational affiliation, years of service) were completed by participants online via RedCap, which is a secure, web-based software platform designed to support data capture for research studies. Qualitative data were then collected through semi-structured interviews (*n* = 3) and focus groups (*n* = 3) conducted by two of the researchers and recorded using a secure, password-protected Zoom software platform. Although recognizing it was not ideal to conduct both focus groups and interviews, we opted to do so to include as many participant voices as possible and in order to stay true to the intention of our data collection processes. Not all PSP were able to attend set focus group times and the researchers did not want to lose the opportunity to speak with all interested PSP. Further hampering data collection was the COVID-19 pandemic, which was in its third wave at the time of data collection (see Ricciardelli et al., 2021 for a description of how COVID-19 has impacted data collection in qualitative research) [80]. Second, focus groups and interviews provided opportunities for specific probing into individual experiences as well as probing into how experiences are expressed across peers (e.g., focus groups encourage participants to build on each other answers; interviews enable probing for clarity). We prioritized focus groups due to our interest in the interactional dynamics between participants but also recognized the need for flexibility brought on by the pandemic. The duration of interviews ranged from approximately 50 to 60 min, whereas the duration of focus groups ranged from approximately 90 to 120 min. The interviews and focus groups were facilitated using a semi-structured guide designed to focus the discussions on preferred delivery modes, psychoeducational training, when such training ought to occur, and for whom it should be tailored for. Interviews were semi-structured and iterative, enabling the researchers to follow a conversational path with each participant. Participants were encouraged to share what they felt was most relevant to each topic explored and probing occurred when clarification was necessary. All recorded interviews and focus groups were professionally transcribed. All researchers and the transcriber signed non-disclosure and confidentiality agreements.

### 2.4. Data Analysis

The interview and focus group data were subjected to thematic analysis, as conceived by Braun and Clarke, using data to identify, analyze, and report patterns (themes) in detail, allowing for the interpretation of various topic aspects [81]. Thematic analysis was used to ensure that primary areas of concern were noted as emergent from the data, in accordance with responses to our questions and the flow of conversation. Such analysis allows for preconceived understanding to fall away and for prevalent experiences to become predominant in how data are interpreted. Thematic analysis also allowed for social and psychological interpretations of data to inform policy development [82]. Practically, thematic analysis involves a detailed inductive and deductive examination of the text to iteratively identify recurring patterns (open coding) that are then refined into ‘themes’ [81]. For this study, both induction (i.e., that which arises from the data naturally and deduction (i.e., that which is related to the research questions) were used.

Data analysis began with two members of the research team familiarizing themselves with the data by reading and re-reading the transcripts while making notes about their initial interpretations. Next the researchers developed preliminary line-by-line in vivo codes (i.e., meaningful labels attached to specific segments of the dataset that are interesting and relevant) [83] and collated the data that were relevant to each code in a systematic fashion. An example of a code was “timing”, as participants frequently spoke about the when they either received or did not receive mental health or resilience training. Once identified, the in vivo codes were moved to a higher level of abstraction by grouping the codes together according to the similarities and differences within and between PSP groups. The primary analyst then developed a coding framework and diagrams (e.g., determining which in-vivo codes fit together and the associated interrelationships) to establish the preliminary themes [84]. Building on the above example, the code “timing” underwent further abstraction and became the subtheme of “too little too late”, as that interpretation seemed more appropriate and specific to the data. Once the preliminary themes were established the researchers used subthemes to support or provide evidence for these larger themes based on their logical relation to the extracts and the entire data set. Once both the themes and the subthemes were created, they were then organized into a coherent narrative that became emergent from responses. For example, the theme of “mode of delivery” was created based on participants repeatedly speaking of their frustrations regarding how training was provided. Next, we coded subthemes of the sources of frustration to support this overall theme. The proposed themes underwent a second round of collective analysis by the research team to capture a more nuanced understanding of the data. This resulted in the researchers reviewing the thematic analysis to look for differences in data interpretations of the analysis. During this review it was clear that the authors’ individual interpretations of the themes were very similar, supporting the overall trustworthiness of the analysis. Further reviews of the data were carried out to confirm that the researchers’ interpretations were identifiable in the original data set. This involved the authors rereading the transcripts after the themes were established, in order to further validate the findings. The excerpts of the participants words illustrate emergent themes and subthemes.

## 3. Results

**Figure 1:**
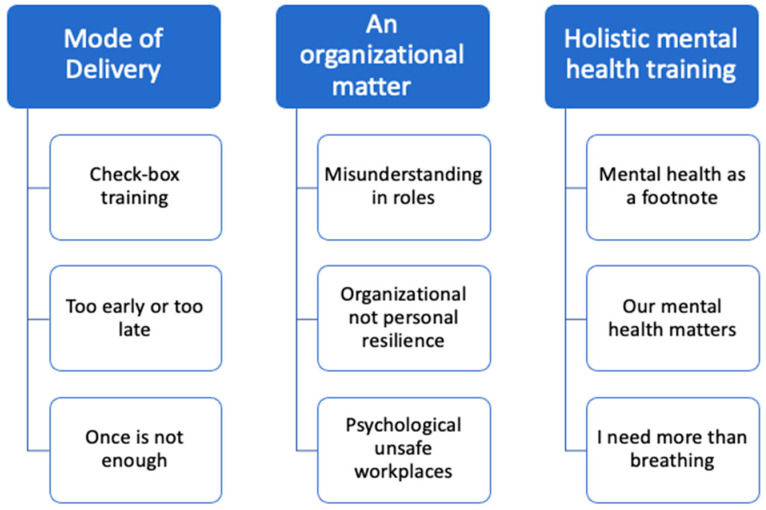
Themes.

The study participants (*n* = 19) included ten firefighters (10 men; mean age 43.3), seven paramedics (four women, three men; mean age 37.6 years), and two public safety communicators (two men; mean age 35.0 years). The thematic analysis resulted in three interrelated emergent themes regarding the delivery for psychoeducational mental health material for PSP: (1) modes of delivery; (2) an organizational matter; (3) holistic mental health training (Figure 1). Participants identified several challenges that were related to the current means of providing mental health education (including moral injury) in PSP, as well as made recommendations to make training more effective and meaningful.

### 3.1. Theme 1: Modes of Delivery

The first emergent theme focused on challenges associated with the current modes of delivery for mental health training and education. Participants described the current modes as being inadequate and often counterproductive to their learning needs. There were three subthemes: (1) check-box training; (2) too early or too late; (3) once is not enough.

#### 3.1.1. Subtheme 1: Check-Box Training

Mental health and resilience training was often deemed to be a requisite exercise on an organization’s checklist of activities. Participants identified the asynchronous online format and lack of specificity to their PSP occupations as the two main reasons for feeling that training was a “check-box”. As one participant commented, “*So when I see training or courses or this or that come in, I see them very much be kind of these little check boxes to meet little mandates or goals (Focus group Paramedic)*”. Participants acknowledged that the asynchronous online format is more cost-effective for broad delivery, but reported that that it required minimal participant engagement with the material (i.e., simply “click through” the material). For example, a paramedic participant expressed:

*I just find that because everything is so online now, that it’s overused. (…) [A]re they actually going to get something out of it or are they just going to play the videos, click through, do something else, do their online shopping, instead of actually paying attention and what they should be doing.*

(Focus group—Paramedic)

The paramedic’s words suggest that attention to the actual training was minimal, perhaps because participants reported that organizations expected mental health and resilience training to be completed between calls for service or during personal time, intensifying the possibility of the training being completed in a rushed and distracted manner—second to their occupational tasks. Public safety communicators underscored the problem, “*we are stuck at a computer overnight and it takes a lot of effort to convince somebody to do continuing education outside of work hours*”. The addition of occupational work to PSP outside of working hours, especially for those confined to a computer during their shift, appears to impact effective learning. Participants also described asynchronous online delivery as lacking specificity or direct applicability to their working environments, which decreased their engagement:

*So I don’t think I’m really into a course. I think we’ve done a lot of courses especially because of COVID-19. I think that the biggest hurdle that all of us have, in my experience, is we’re looking for help and we’re looking for someone that could understand us because we are different from civilians.*

(Focus group—Firefighter)

As reflected by this participant, PSP training needs being recognized as unique is best received from facilitators who are familiar with PSP exposures and/or who have had similar experiences. Some participants wanted training to be in person, whereas others wanted remote digital options to support anonymity, particularly when discussing personal topics such as mental health. Across delivery mode preferences, participants described the level of engagement, applicability, and interest as driving their commitment to training.

#### 3.1.2. Subtheme 2: Too Little or Too Late

Most participants reported having received minimal mental health training, either extremely early in their careers (i.e., during their schooling or when they were first employed), or significantly later in their careers when seeking out additional mental health training or resilience education, either as a part of working towards career advancement or in trying to relieve significant mental health challenges that had developed during their career. Participants highlighted the need for regular training that is tailored to different career stages:

*I think that we need to be recruiting for that a bit more and it [mental health or resilience education] needs to be a focus way early on and it needs to be embedded in our education and then it needs to be actual robust support and then you need to have that ongoing continual continuing education like these types of pieces of maintenance*.

(Interview—Paramedic)

The paramedic’s words reinforce that training early in occupational tenure may be less effective, largely because of a lack of experience—PSP are thought to not fully value the training because they have yet to see the occupational impacts of their operational responsibilities. Participants shared that not having the lived experience early on in their careers to fully grasp the significance of what was being taught was problematic.

*I would suggest it may be helpful to do at school for a Paramedic, or Fire, or Police because going back to what XX is saying about not really knowing what you’re walking into. I don’t recall touching on anything psychological in PCP or Fire Fighting. I don’t know, earlier before you start the job maybe it would be beneficial going to school.*

(Focus group—Firefighter)

The participant’s words echo the need for regular psychological education that begins in training and continues throughout their careers. Regarding moral injury, participants reported that their core values had changed significantly during their careers as a result of exposure to occupational stressors. Participants emphasized the need for regular personal reflection on their emotional and psychological health (e.g., once per year) throughout their career:

*From a user perspective you know, almost like and it really should be ongoing, almost a self-checklist of key emotions that some people might be feeling that might be triggers that maybe they’re having some issues that would need to be addressed… Yeah, some kind of a self-check that they would do periodically would be important for them.*

(Interview—Public Safety Communicator)

Participants emphasized the desire for ongoing mental health training from people with shared experience or awareness of the challenges faced by PSP. Greater knowledge about the realities of PSP work and how PSP work experiences can affect mental health, particularly when presented during initial training, was seen as valuable aspect of psychological resilience.

#### 3.1.3. Subtheme 3: Once Is Not Enough

Participants reported that most psychoeducation was focused on the effects of PPTEs. Participants criticized program-based, one-off approaches to mental health as demonstrating leaders’ lack of awareness of the impacts of chronic and cumulative exposures to diverse PSP occupational stressors.

*Because I sit there on the receiving end of ‘here’s your other mandatory, here’s your other R2MR, here’s your this, here’s your this, here’s your this.’ … what I can’t echo … enough is … the focus of this learning and this education has to be system level. Once is not enough…*

(Focus group—Paramedic)

*I think on our leadership side of things regarding mental health, there is a problem. They [leaders] could say, “we gave you that moral injury course.” So they check it off in their box and they know that they’ve done their job and that’s all they… It just kind of stops at that. But on their side that’s all their, “well we gave you this course, so now we’re good.” You know what I mean?*

(Focus group—Firefighter)

The participants’ words evidence that individual level training falls short of protecting their mental health, and that a cultural change toward making the workplace environment a psychologically safe space is warranted. Some training and mental health programs were perceived as meaningful; however, participants underscored that focusing on PPTE as the only mental health risk was extremely out of touch with contemporary PSP needs:

*We do have things in place where the critical incidents are documented. The Call Takers reaction is documented and that is supposedly taken up the chain to our direct manager and I’m sure in some cases they’re followed up on but I don’t have any good examples of that.*

(Interview—Public Safety Communicator)

Even though processes are in place to document exposure to PPTE, participants reported feeling unclear on how the process worked or whether the process was actually beneficial for them. Several participants reported wanting a systematic approach to mental health in the workplace that did not bestow the accessing of mental health support as being the sole responsibility of individual PSP. Participants highlighted that PSP experiencing mental health challenges may not have the capacity in the moment to engage mental health resources when needed.

*It’s like if you go fishing, you’re not going to expect a fish to jump in the boat. You can’t go fishing and be like, “where are all our fucking fish? They’re not jumping in the boat for us.” I hear that from so many different organizations. Like we have the CISM program. We have this program. We have EAP programs. We have all this stuff. What else are we supposed to do? I’m like, “oh my God. If that’s your attitude then…”*

(Focus group—paramedic)

Participants emphasized the need for an organizational culture where mental health is the responsibility of everyone, not just those who suffer.

### 3.2. Theme 2: An Organizational Matter

The question of who should receive mental health training and for what purpose emerged as a second primary theme. There were three sub-themes indicating mental health programs have been inordinately focused on individuals rather than organizations or organizational culture. Sub-themes included: (1) the misunderstanding of roles; (2) organizational instead of personal resilience; (3) psychologically unsafe workplaces.

#### 3.2.1. Subtheme 1: Misunderstanding of Roles

Participants identified the perceived lack of leadership training at all levels as a primary problem for supporting mental health and resilience. There is increased messaging that frontline PSP should access their supervisors for mental health support, but frontline PSP and middle managers felt that training was lacking.

*I spoke to this earlier…To mention the leadership course…That was just a series of questions that they get you to answer, then you tally a score, and then you take that score, and you do some circle pie chart that you scratch off different things. And then the instructor would say, “do not go ahead. Only do this step…”*.

(Focus group—Firefighter)

The firefighter’s words suggest a restrictiveness in completing courses that emerges from a disconnection, perhaps even distrust, of supervisors. Participants described negative interactions upon approaching supervisors about mental health concerns, including judgments and biased opinions about the employee, such as “*oh they’re just not cut out for EMS. They can’t handle it. They can’t cut it*”. *And part of it is, is this actually true? Or are we just breaking them? Are we eating our young in this case? Or our old?* (Focus group—Paramedic). One participating manager acknowledged the problem by describing feeling unqualified to address employee mental health challenges, but being responsible for providing meaningful help:

*I think somewhere along the line, somewhere in some people’s minds, the supervisors, management, or anybody in a leadership position have some special, special training that we don’t. Or even how to deal with a person in crisis. I don’t have any training for that. Nor do I think I should. I should have a good idea of what resources are available to us to help us. Um, and to help identify when someone’s in crisis I guess, but even that is not the end-all, be-all, right?*

(Interview—Public Safety Communications Manager)

The manager emphasizes the need for specialized training to help identify employees in crisis and for clarifying the role of a manager in crisis situations. Even without such training, the hierarchical nature of PSP organizations and the associated promotion processes were identified as being problematic for mental health. Participants reported variability among leaders, but those leaders were often selected because of seniority, years of service, or nepotism, instead of leadership qualities and mental health awareness.

*“[T]here’s a difference between leadership and managers and we have a collection of managers, there is a beautiful number of leaders throughout our profession they just may or may not have that rank on their shoulder and therefore don’t have the same type of influence and power connected to it”.*

(Focus group—Paramedic)

Participants viewed the selection of leaders as varying, distinguishing between leaders and managers. Participants described managers as bureaucrats who were more interested in appeasing the system than acting in the best interest of employees. Participants spoke highly of leaders who, they envision, do right for their people regardless of the challenges or barriers. Sadly, participants’ interaction with those leaders were few and far between.

#### 3.2.2. Subtheme 2: Organizational, Not Personal, Resilience

Mental health education for resilience training continues to focus on individual PSP rather than the organization as a whole. One participant commented, “*I think there’s all these courses like the R2MR and resiliency for firefighters and all these courses that I think they’re great, but I think it’s kind of like putting a Band-Aid on a broken leg” (Paramedic).* Participants also shared frustration over a continued focus on individual PPTE, despite evidence that diverse occupational stressors, particularly preventable organizational stressors, often contribute to mental health challenges [9].

*If the person experiencing the problem goes to leadership and they say “okay definitely take the rest of the day off, go for a walk, go for a jog, eat something good because XYZ”… That is going to go a long way to translating to people actually doing the things.*

(Interview—Public Safety Communicator)

Unhealthy organizational realities, such as concern about leadership abilities and quality, were described as having significant negative impacts on participants’ treatment-seeking as well as their feelings of support. Participants emphasized that mental health and resilience training need to be viewed from a social-ecological lens, challenging organizations to consider their business practices and efforts to foster a culture of respect, dignity, and psychological safety. One participant reported being required to adopt a trauma-informed approach when caring for their patients, but that their organization failed to take a trauma-informed approach to operations.

*Fixing the culture and mindset and the system of EMS would fix so much more of the retention issues and everything than just a little education at the beginning because you can introduce all of these courses and you’ll be a fresh-faced young paramedic and you’ll work with a 20 year paramedic that looks at you and is like, “Oh look at you and your nice thoughts about people actually being people. Let me show you how it really is.” These guys are animals. Okay… you need help.*

(Interview—Paramedic)

Participants appeared to want a comprehensive approach that addresses the organizational culture, in order to positively inform employee wellness.

#### 3.2.3. Subtheme 3: Psychologically Unsafe Workplaces

PSP participants reported how problematic and toxic their workplaces had become as a result of poor leadership, culture, or values, and a lack of effort to address systemic issues within their own organizations.

*Moral injury…shows up in my workplace far more from the work culture environment than the calls themselves. And it shows up personally for me from everyday sexism at work from watching my peers experience that*

(Focus group—Paramedic)

While PSP are expected to treat patients with dignity and respect, the same expectations are evidently not reflected between employees in the workplace. Mental health stigma is still apparent in the PSP workplace as participants indicated that being vulnerable or expressing genuine feelings often translated to being perceived as weak. Rather than working with a co-worker who is struggling, this person is, too often, “written off”. One paramedic exemplified:

*I’ve been told this to my face. “Look, if these people are too weak to work here, I don’t want them here.” So that attitude is still there. … [A]nd it happens here in XXX, where paramedics … or fire fighters are getting pushed out because the employer doesn’t want them back because they feel they’re too weak*

(Focus group—Paramedic)

The paramedic underscores the impact of stigma on help-seeking, a very real stigma that appears engrained in organizational culture. Participants reported often lacking the necessary skills to address challenges such as confronting a struggling coworker or a hostile boss. Nevertheless, participants also acknowledged the positive impact that strong leaders had on their mental health and overall well-being when attempting to address systemic issues.

*Leadership here in XXX right now is probably the best we’ve ever seen and right now would be the perfect time to, I don’t know… implement something because these guys understand. They’ve been on the floor; they understand how things work and how things are running. … those guys get it.*

(Focus group—Firefighter)

As evidenced in the excerpt from the firefighter, there is much to be gained from supportive leadership, which can also positively inform help-seeking behaviors by creating and ensuring a supportive and compassionate environment.

### 3.3. Theme 3: Holistic Mental Health Training

Previous research has evidenced participants reporting that learning technical skills that are required to perform emergency lifesaving and rescue procedures was the primary focus of their pre-employment training [7]. All participants commented on a lack of training in the “soft skills” (e.g., morals, ethics, values, character, communication, social interaction skills), resilience, and mental health knowledge required to effectively perform their occupational duties. Participants described the training gap as placing them in situations where they felt underprepared or helpless, particularly when trying to help patients or callers experiencing psychological distress or mental health challenges. PSP also reported being provided even less training about their own mental health, how mental health challenges may present among coworkers or organizations, or what to do about mental health challenges. Sub-themes included: (1) mental health as a footnote, (2) mental health matters, and (3) participants needing more than just breathing exercises.

#### 3.3.1. Subtheme 1: Mental Health as a Footnote

Participants described receiving only minimal education and training (approximately 3 h) about mental health. Participants reported learning relatively little about assessments, interactions, and the provision of care for patients with systemic socioeconomic and behavioral challenges (e.g., homelessness, addiction, domestic violence).

*And there’s like domestic violence. Where the woman goes back to… I go to the same people all the fucking time and it’s like he beats the shit out of her and he’s like, “but we love each other.” And you just want to take him and beat the shit out of him. She’s got two black eyes now and you’re just like, “why the fuck don’t you leave this situation?”*

(Focus group—Paramedic)

Paramedics and firefighters reported feeling underprepared to support patients with mental health challenges and reported frequently having to learn what to do from colleagues or by trial and error.

*How do I stay respectful to this intoxicated, clearly homeless, hasn’t washed in a very long time and smells terrible. How do I treat that person with the same care and respect as the 65-year-old grandmother I go to later who fell and broke her hip… And then we get the moral injury afterwards and I feel like a lot of it is self-inflicted because if we don’t learn it in school, we learn it from our preceptors, we learn it from our coworkers*

(Interview—Paramedic)

Public safety communicators reported having no training on mental health or how to communicate with a caller who was agitated or panicked because of a serious incident. One participant spoke of the absent training in terms of the difference between PPTE and other highly emotive stressors.

*“Well, I’m very happy that we do have such an accepting process or an accepting stance on critical incidents but then um, the difference being having eight kind of rough ones, that’s basically the job. You know, you don’t sign up for pediatric cardiac arrest, but you do sign up for taking eight overdose calls”.*

(Interview—Public Safety Communicator)

The perceived training gaps appear to place a significant burden on all PSP; even earnest attempts at mental health training and education is never more than a footnote.

#### 3.3.2. Subtheme 2: Mental Health Matters

PSP criticized training failures with respect to the mental health impact of occupational stressors, ranging from the benign to the absurd and horrific. In particular, PSP wanted their organizations to understand that their mental health matters as much as that of their clients.

*So, then I talk to the frontline guys and they say, “Yeah well, they say that they’re priority is mental health, but it seems like they just want to appear that way then actually doing stuff about it.” And I find that’s very common. So, I think, you know, it’s important for the idea or the concept to appear that their service has mental health as a priority that in some cases is more important to them than actually making the priority or prioritizing mental health.*

(Interview—Paramedic)

Participants described feeling frustrated by a tangible lack of organizational support despite the attention directed at PSPs’ mental health. Consequently, many participants reported feeling hypocrisy regarding what they were being told versus what they perceived as truly happening on the ground. PSP often reported being left to naively self-manage the psychological effects of their occupational stressors. Left unresolved, participants described repeatedly experiencing feelings of helplessness that often became sources of frustration, cynicism, jadedness, anger, hostility, burnout, and PTSD.

*I don’t even think they’re doing okay with PTSD. I think we’ve just rounded a curve of attention on it. I will maybe give credit for attention on it, beyond that, the jury is out.*

(Focus group—Paramedic)

The frustrations were compounded when PSP looked to their organizations or peers for help with psychological stressors or distress only to find that the organizational and peer resources were late, lacking, or problematic (i.e., their peers were as deeply wounded as the person looking for help). Participants explained:

*So if you actually… it’s one of two ways, either A) Just kind of practice what you preach or just tell us the truth that, “Hey, we believe in mental health, but we sure as shit aren’t going to fund it.” That’s fine. Just be honest with us because the disconnect, it’s the problem.*

(Focus group—Paramedic)

*But the phone never stops. That workload doesn’t stop. So the answer some people will say is, “[W]ell just tell them to just take them out and have a talk with them for half an hour.” Haha.*

(Interview—Public Safety Communicator)

In the first excerpt, the participant spoke to the need of organizations to be honest with their employees about the vocational realities, including when to ask for mental health help. In the second excerpt, the participant exemplified that, despite best intentions (e.g., having communicators take a break), the realities of the phone never stopping made such practices impossible. As such, the solutions and realities were largely incongruent. Participants identified the need for organizational training that adequately and comprehensively acknowledges the probable effects of PPTEs, PMIEs, or other occupational stressors inherent to PSP work.

*And it can’t be really fixed with a course. … [E]veryone has different morals and experiences; it needs to be more specific. I mean, courses will help, but it’s not going to fix us. I guess it’s education versus fixing. I don’t think it’s going to fix anyone in these situations. It might help educate myself, help my peers, or educate what’s going on inside but that’s all….*

(Focus group—Firefighter)

Overall, participants noted a disconnect and perceived hypocrisies between suggested strategies to support their mental health and the lived realities of their work.

#### 3.3.3. Subtheme 3: I Need More Than Just Breathing Exercises

Participants emphasized the importance of mental health education being more in-depth and more applied. While acknowledging the importance of training in basic coping skills such as breathing or meditation, participants criticized the singular focus on mental health literacy as lacking relevant and effective skills supported by practice with help from experts.

*We had to take R2MR, which is fine. It’s good information, however, it was nothing new. Nothing we don’t know. All it did was give us a name. So now when we’re angry instead of just being angry we start yelling, “I’m in the red! I’m in the red!” So it kind of became a running joke because yes, we know your partner is in the orange, we know you’re in the red, but it never gave anything for us to do.*

(Focus group—Paramedic)

The participant laments the lack of applied skills tied to the training they have received. Another participant reported that many PSP have enough knowledge to recognize that they are unwell, but have little information about what to do next other than call the Employee Assistance Program.

*We already have a program where certain employees have volunteered to be points of first contact and those employees… Yeah, they’re the ones that tell you to breathe, eat some soup, have a run, and then give you a phone number to call.*

(Interview—Public Safety Communicator)

The communicator’s words suggest that interventions and available support are lacking or far from practical. Accordingly, participants expressing a desire to receive ongoing, progressive, in-person, interactive training facilitated by an expert should not be surprising. Participants also expressed a desire for more intensive simulation training, which would focus on different external mental health scenarios as well as explicitly focus on the impact of PPTEs and PMIEs, and how to help support struggling coworkers. Participants reported that this type of intensive real-life mental health training might help to mitigate the fear, lack of knowledge, powerlessness, and stigma associated with the exposure to occupational stressors.

*We did some CISM training with the police and our experiences are pretty much similar…. Um, it was way better than taking CISM Training from someone who had never worked in the First Responder field. Because I think that courses were helpful, and I thought it was just so much better when the police did it because they had personal stories and they had examples of real-life situations… rather than just reading from a book.*

(Focus group—Firefighter)

The need for mental health training that is led by PSP, for PSP, or by an expert familiar with the population, was noted as being potentially highly beneficial because of the ability to integrate real scenarios and lived experience. Participants also shared that current approaches which focus on mental health education miss the need for training that provides concrete and immediately accessible strategies that PSP can use within the field.

## 4. Discussion

We designed the current study to help clarify PSP preferences regarding the modes of delivery, training frequency, and content for psychoeducational material. The results suggest that firefighters, paramedics, and public safety communicators feel that contemporary approaches to improve mental health and resilience during their pre-job training and in their workplaces remain lacking or nonexistent. Participants reported a lack of preparedness for dealing with personal mental health problems and discussing mental health with co-workers. Participants also described the available organizational mental health supports as being questionable. The provision of sporadic one-off mental health and resilience programs by organizations was considered welcome, but insufficient. Participants reported needing more engaging methods for delivering information, career-long mental health knowledge acquisition, and a systems approach to improve workplace culture, particularly that pertaining to mental health.

Participants expressed interest in a mixed approach to workplace mental health training, describing online training as often being an exercise in ticking boxes instead of being an engaging process to help solve problems; mental health was described as a “footnote” rather than a priority. Face-to-face training is expensive because of direct program costs (e.g., trainers, materials) and the indirect costs of back-filling staff who are away being trained [77]. For PSP organizations, face-to-face training may be particularly problematic because of insufficient human resources, interacting with mandated minimum staffing levels [85]. Participants underscored difficulties with not being allocated work time to participate in mental health or resilience training. The unpredictable workday demands of PSP service make allocating training time during work hours difficult and expensive, potentiating training being required during unpaid hours, which can facilitate resentment, decrease engagement, and compromise effectiveness [86].

Digital mental health training has demonstrated statistically significant effects post-intervention on both psychological well-being [32] and work effectiveness when compared with control conditions [86]. Digital mental health and resilience training may also be appealing to organizations as a relatively inexpensive and convenient way to administer workplace training. The approach, however, appears overly simplistic for managing the complexities associated with mental health. Singular asynchronous efforts result in higher rates of dropouts and incompletions for psychotherapeutic training [18]. The flexibility and lack of monitoring in digital training can also inhibit engagement, particularly if there is not enough work time allocated to learners [86]. Guidance, adjuvant communication modalities (e.g., texting, emailing), and persuasive technologies (e.g., self-monitoring or tailoring to the specific workgroup) can all increase training engagement and adherence [86]. Participants reported valuing the anonymity and convenience of digital training but wanted to also have interactive and in-person training with occupation-specific information, provided by culturally aware mental health professionals and peers.

Increased engagement through multimodal [18] or multiple training sessions may improve the sustainability of the short-lived changes in resilience and stigma reduction [35,37]. The evidence available suggests that longer programs do not produce better results [26], but distributed sessions repeated over time may elicit better outcomes [35,37]. The effectiveness of multi-modal experiential and high-fidelity scenario-based training that integrates psychoeducation is being explored [87,88] and may better facilitate the integration of key learnings and tools into practice, particularly for PSP. Experiential learning can be a holistic adaptive process of learning that merges perception, cognition, and behavior and experiences, where learners construct knowledge and meaning from real-life experiences [89]. Giving PSP the opportunity to practice and receive feedback on their use of mental health skills should support skill improvements and confidence, which should ultimately improve their mental health.

The participants and existing research support the notion that occupational stressors other than PPTE (i.e., organizational stressors like culture, workload, infrastructure, teamwork) can account for substantial variance in mental symptoms [9,90] and can negatively impact wellness [7,91]. Accordingly, the focus on addressing exposures to PPTE and PMIE to improve PSP mental health may be beneficial by expanding it to include other occupational stressors [9]. Current resilience training focuses on individual frontline PSP, with no or little emphasis on organizational changes that may offer important opportunities for increasing PSP psychological resilience and well-being [9,30,31]. Focusing on individual stress-management can help workers build resilience skills, but it remains unlikely to maintain long-term employee mental health without organizational procedures for reducing or preventing systemic workplace psychological stressors [92,93].

Understanding that the individual is an occupant of an organizational role, as well as a human who is potentially vulnerable to poor mental health, can empower leaders to create cultures of care, safety, and training. PSP leaders who actively and regularly use evidence-informed training in mental health can create psychologically safe workplaces that are inherently more resilient and better able to protect frontline PSP [94]. Systemic organizational changes may alleviate job stress and maintain the wellness of employees as part of proactive efforts to mitigate mental health challenges, by facilitating psychologically safe workplaces and culture change [9,30]. Creating an organization of safety, trust, and open communication can help to facilitate improved mental health, increase uptake of mental health training, and improve employee performance and organizational commitment [95,96].

The current study has several limitations that offer directions for future research. First, the analysis was derived from the experiences of Canadian firefighters, paramedics, and public safety communicators, which makes generalizability to all PSP populations unlikely. Like all qualitative research, any generalizability warrants caution. Second, the broad inclusion criteria meant that participants represented a spectrum of positions within their respective occupations, with varying experiences and years of service (e.g., most participants had served between 10 and 15 years as a PSP). Participant variability, however, was not distinguished in the current data analysis. Third, participants were recruited via snowball and convenience sampling which may have biased the data to self-selecting individuals who were interested in the study or who wished to have their opinions heard. Fourth, recruitment occurred during the COVID-19 pandemic, which may have impacted the ability to recruit participants and could explain why the recruitment of police officers during the study period was unsuccessful. Conducting this research during the pandemic may have also influenced the provided data because of the intensity and reported struggles associated with PSP service during the pandemic.

## 5. Conclusions

The current results provide important information about PSP preferences regarding modes of delivery, training frequency, and content for psychoeducational material. Participants indicated current mental health training programs would benefit from more active engagement, more frequent and tailored presentations from initial training through to retirement, and more evidence-based skills that directly impact mental health challenges. The participant feedback suggests that a critically constructive review of mental health training may provide additional insights for iterative improvements in its effectiveness. Clear, accepted definitions for resilience, interactive training, and applied attention to occupational stressors beyond PPTE and PMIE may all be tangible pathways to support improvements in mental health training. PSP leaders may also benefit from dedicated training that provides tools to meaningfully support the mental health of frontline PSP. Participating PSP appear to want systemic-evidence-based organizational changes to promote psychological wellness. Future research on improving PSP mental health should include a focus on implementation science to support pervasive change.

## Data Availability

Data available on request only due to privacy/ethical restrictions. The data that support the findings of this study are available on request from the corresponding author, L.S.-M. The data are not publicly available due to their containing information that could compromise the privacy of research participants.
